# Long-term outcomes of genome-edited “universal” CAR19 T cells for relapsed/refractory B-ALL at a single pediatric center^∗^

**DOI:** 10.1182/bloodadvances.2025016366

**Published:** 2025-07-03

**Authors:** Daniela Guardo, Avijeet Kumar Mishra, Hebatalla Rashed, Kimberly Gilmour, Stuart Adams, Danielle Pinner, Martin Sauer, Ajay Vora, Paul Veys, Vesna Pavasovic, Kanchan Rao, Waseem Qasim

**Affiliations:** 1 Department of Bone Marrow Transplant, Great Ormond Street Hospital for Children National Health Service Trust & University College London Institute of Child Health, London, United Kingdom; 2 Department of Bone Marrow Transplant and Cell Therapy, Hannover Medical School, Hannover, Germany

**Figure:**
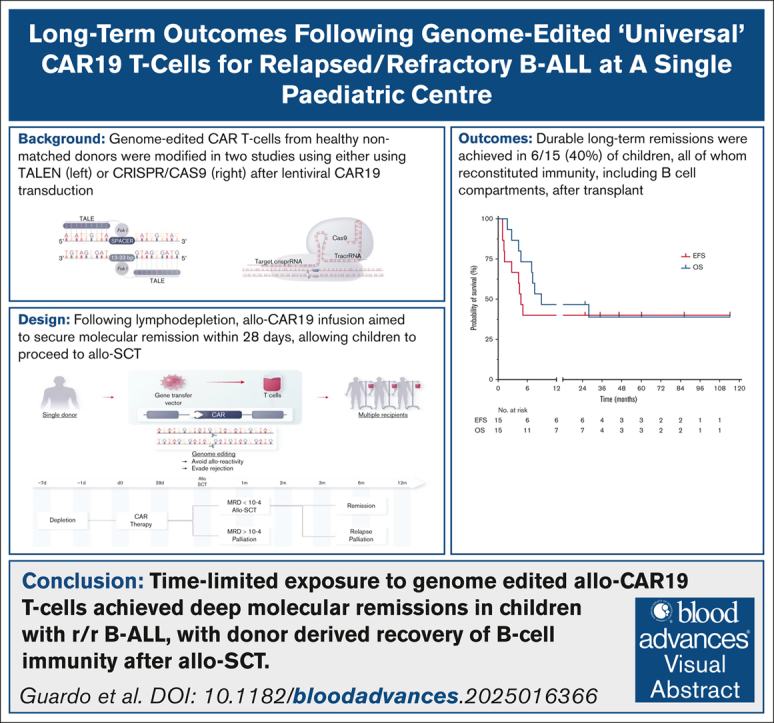

**TO THE EDITOR:**

Although survival outcomes for standard-risk pediatric B-cell acute lymphoblastic leukaemia (B-ALL) are above 90%, high-risk refractory/relapsed (R/R) B-ALL requires additional therapeutic avenues.^1^^,^^2^ Immunotherapy using bispecific T-cell engagers, such as blinatumomab,^3^^,^^4^ or autologous anti-CD19 chimeric antigen receptor (CAR19) T-cell therapies offers promising alternative strategies.^5^, ^6^, ^7^ In addition, allogeneic “off-the-shelf” CAR19 T-cell therapies are becoming feasible through genome-editing strategies and could help address manufacturing and logistic hurdles to unlock timely and cost-effective interventions for larger number of patients.^8^

At Great Ormond Street Hospital, we adopted transcription activator-like effector nuclease (TALEN) as part of early strategies to disrupt T-cell receptor (TCRαβ) for prevention of graft-versus-host disease (GVHD) and included CD52 knockout to promote persistence of donor-derived CAR T cells in the presence of alemtuzumab, an anti-CD52 antibody deployed to augment lymphodepleting effects of fludarabine and cyclophosphamide. Successful compassionate use experience with TALEN-edited CAR19 T cells in 2 infants with R/R B-ALL^9^ was followed by further treatment of 6 children as part of a phase 1 clinical trial of UCART19 (ClinicalTrials.gov identifier: NCT02808442),^10^ and an additional 7 children were subsequently treated with TT52CAR19 T cells (ClinicalTrials.gov identifier: NCT04557436), donor T cells edited using Clustered Regularly Interspaced Short Palindromic Repeats (CRISPR)/Cas9 technology.^11^ Both trials delivered a “time-limited” exposure to engineered T cells for 28 days before bone marrow reassessment, and then, if in remission, the children proceeded to allogeneic stem cell transplantation (allo-SCT) between days 41 and 79. These approaches aimed to secure deep molecular remission of leukemia before donor-derived reconstitution of all immune compartments through allo-SCT. Building on reports from autologous experiences,^6^^,^^12^ we captured survival, disease status, and immune reconstitution for all children who have received allogeneic genome-edited CAR19 and then proceeded to allo-SCT.

Outcomes were surveyed for children (aged under 16 years) with CD19^+^ R/R B-ALL who were managed as part of phase 1 studies or under compassionate-use arrangements. Between 2015 and 2022, 15 high-risk children, with a median age of 2.5 years (range, 0.8-16.6), received either UCART19 (n = 2, compassionate; n = 6, NCT02808442) or TT52CAR19 (n = 7, NCT04557436). Ten patients had infant B-ALL (67%), and the most frequent rearrangements involved *MLL* (73%). Most (60%) patients were in their second or subsequent relapse and had undergone a variety of additional interventions, including previous allo-SCT (n = 10), blinatumomab therapy (n = 5), or autologous anti-CAR19 T-cell dosing (n = 3) (Table 1).

**Table 1.** **Patient characteristics and procedures**

Patients	S1	S2	P1	P2	P4	P5	P6	P8	TT2	TT3	TT5	TT6	TT7	TT8	TT9
Disease	Infant ALL	Infant ALL	Pre-B ALL	Pre-B ALL	Pre-B ALL	Infant ALL	Pre-B ALL	Infant ALL	Infant ALL	Infant ALL	Infant ALL	Infant ALL	Infant ALL	Pre-B ALL	Infant ALL
Genetics	MLL	MLL	ETV6-RUNX1	MLL	Tril t(2;17)	MLL	47 XX, gain c18/X	MLL	MLL	MLL	MLL	MLL	MLL	ETV6-RUNX1	MLL
Previous SCT	Y	Y	N	N	Y	N	Y	N	Y	Y	Y	Y	Y	N	Y
Previous CAR19	N	N	N	N	N	N	N	N	N	Y	N	Y	N	Y	N
Previous BiteY	Y	Y	N	N	N	N	N	N	N	N	Y	N	N	Y	Y
Disease burden ∗PCR	12%	10%	80%	7%∗	3.4%	11%	1.3%	1%∗	3.2%	21%	66%	0.6%	3.2%	58%	0.9%
Lymphodepletion	F90 C120 A1	F150 C120 A1	F150 C120 A1	F150 C120 A1	F150 C120 A1	F120 C120 A1	F150 C120 A1	F150 C120 —	F150 C120 A1	F150 C120 A1	F150 C120 A1	F150 C120 A1	F150 C120 A1	F150 C120 A1	F150 C120 A1
CRS	N	N	G ≥3	G2	G2	G1	G1	G ≥3	G1	G1	G1	G1	G2	G2	G2
ICANS	N	G ≥3	N	N	N	N	N	G1	N	N	N	N	G ≥ 3	G1	N
Viral comps.	N	N	N	CMV ADV	N	N	BK	N	CMV	ADV	N	ADV	N	N	N
D28 Flow	ND	ND	NEG	NEG	ND	ND	ND	POS	ND	POS	NEG	POS	ND	NEG	ND
D28 PCR	NEG	NEG	POS	POS	NEG	NEG	NEG	POS	NEG	POS	NEG	POS	NEG	POS	NEG
Allo-SCT	Second MMUD RIC	Second MUD RIC	MUD MAC	MUD MAC	Alt MSD MAC	MUD RIC	Second MMUD RIC	N	Second MSD RIC	N	Second MUD RIC	N	Second MUD RIC	MUD MAC	N
Chemotherapy	F,C, Thio	F,C	C	C	C	F,T, Thio	F	N/A	F,C	N/A	F,C	N/A	F,C	E	N/A
ATG	Y	Y	Y	Y	N	Y	Y	N/A	N	N/A	Y	N/A	Y	N	N/A
Radiotherapy (Gy)	4	2	14.4	14.4	14.4	0	2	N/A	2	N/A	2	N/A	2	12	N/A
FU and outcome	AW	AW	DRM	DRM	TRM	AW	AW	DRM	AW	DRM	AW	DRM	DRM	DRM	DRM

A, alemtuzumab; ADV, adenovirus; Alt, alternative; ATG, antithymocyte globulin; AW, alive and well; BK, BK virus; C, cyclophosphamide; CMV, cytomegalovirus; CRS, cytokine release syndrome; DRM, disease-related mortality; E, etoposide; F, fludarabine; FU, follow up; G, grade; ICANS, immune effector cell–associated neurotoxicity syndrome; MAC, myeloablative conditioning; MMUD, mismatched unrelated donor; MSD, matched sibling donor; MUD, matched unrelated donor; N, no; N/A, not applicable; Neg, negative; ND, not determined; Pos, positive; T, treosulfan; TRM, treatment-related mortality; Thio, thiotepa; Viral comps., viral reactivation in the first 28 days after allo-CAR19; Y, yes.

∗ Disease burden was quantified by flow cytometry unless by PCR where indicated.

Both investigational products used a self-inactivating lentiviral configuration encoding an anti-CD19 binder, 41BB, and CD3z chimeric receptor under the control of a human phosphoglycerate kinase promoter. Augmented lymphodepletion comprising fludarabine 150 mg/m^2^, cyclophosphamide 120 mg/kg, and alemtuzumab 1 mg/kg (total doses) was used in 12 of 15 recipients. Two of the youngest infants (patients S1, P5) received reduced fludarabine (90-120 mg/m^2^), and alemtuzumab was omitted for 1 UCART19 recipient (patient P8). Doses of allo-CAR19 T cells infused ranged between 1.1 × 10^6^ and 4.6 × 10^6^ T cells per kg for UCART19 or 0.8 × 10^6^ and 2.0 × 10^6^ T cells per kg for TT52CAR19, and TCRαβ cell carriage was capped at <5.5 × 10^4^ per kg.

Complications included cytokine release syndrome (CRS) (grade 1-2, 73%; grade 3-4, 13%), immune effector cell–associated neurotoxicity syndrome (grade 1, 13%; grade 3, 13%), and viral reactivations (33%). Bone marrow assessments after 28 days revealed that 11 of 15 (73%) patients had achieved complete remission with or without count recovery with undetectable leukemia by flow cytometry. Quantitative polymerase chain reaction (PCR)–based corroborated remission as minimal residual disease (MRD)-negative (<0.01%) in 8 of 15 (53%) patients (Table 1). Allo-SCT was undertaken within a median of 2.1 months (range, 1.9-3.1) after allo-CAR19 T-cell infusion, mainly using reduced intensity conditioning (RIC) with antithymocyte globulin and low-dose total body irradiation (TBI) (2-4 Gy). Furthermore, 6 of 7 were second transplant procedures using the original stem cell donor. The remaining 4 subjects received myeloablative transplants (TBI, 12-14.4 Gy) with 3 of 4 as first transplants from matched unrelated donors.

**Figure 1.**
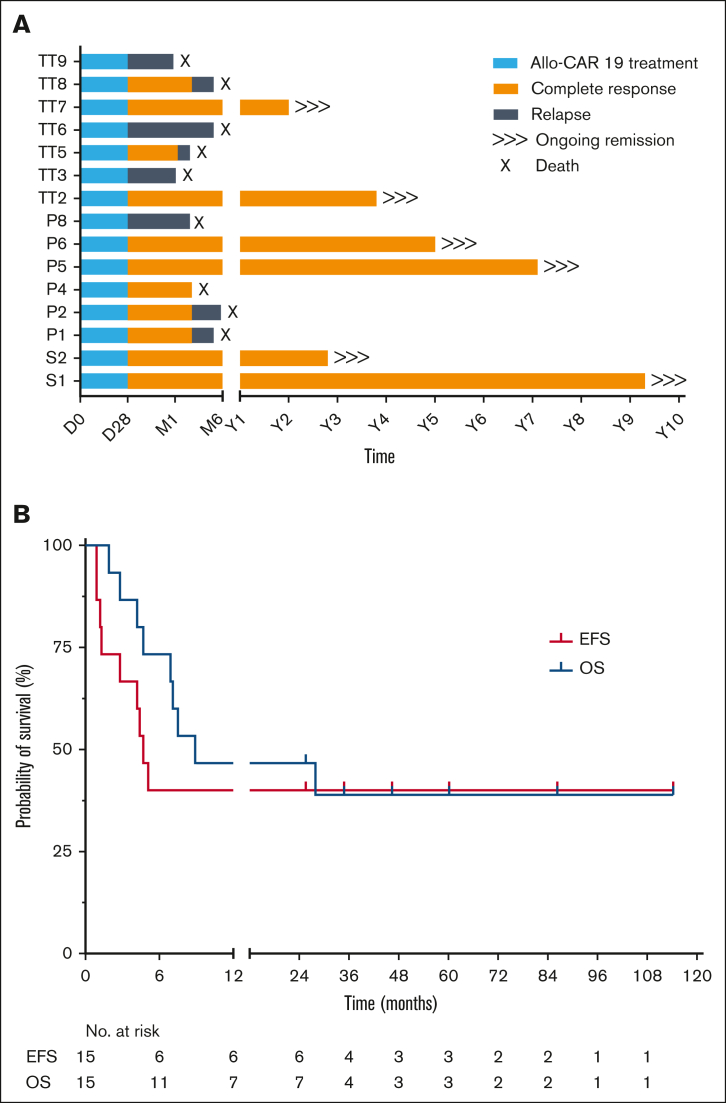
**Survival outcomes.** (A) Outcomes for all children treated at Great Ormond Street Hospital with genome-edited allo-CAR19 T cells. Periods of allo-CAR19 intervention (blue), complete remission (orange), or relapse (gray) are illustrated after 2 to 10 years of follow-up. (B) EFS and OS with 40% of subjects in long-term remission above 2 years. EFS, event-free survival; OS, overall survival; P, trial UCART19 (P); S, specials license UCAR19; TT, trial TT52CAR19.

Molecular remission was sustained in 7 of 8 children (88%) who underwent transplantation but were MRD-negative by quantitative PCR (<0.01%). Three patients who underwent transplantation with flow-negative MRD, but detectable PCR-MRD, all relapsed after the transplant. Acute GVHD was limited to grades 1 to 2, and no chronic GVHD was reported. After conditioning and allo-SCT, nonpersistence of allo-CAR T cells was confirmed in all patients by chimerism and vector copy number quantification. Viral reactivations were monitored and treated with antiviral drugs until reconstitution (Table 1; supplemental Figure 1), although 1 subject (P4) died from complications associated with symptomatic BK virus and microangiopathy, which developed after SCT and while in remission. At median post-SCT follow-up of 4 years (range, 2-10), 6 subjects remained disease-free with overall event-free survival of 40% (6/15) (Figure 1). Of note, all survivors had undergone RIC transplants and exhibited normal immune recovery after allo-SCT. T-cell and B-cell counts recovered by ∼6 months (4.3-12.2) (supplemental Figure 2) allowing withdrawal of IV immunoglobulin replacement therapy where it had been initiated.

Genome-edited allo-CAR T-cell strategies are being investigated in early phase trials as alternatives to autologous CAR therapies. Various platforms can disrupt T-cell receptor αβ (TCRαβ) expression and unlock strategies to evade host immunity either directly (by HLA disruption) or indirectly, by conferring resistance to agents such as alemtuzumab.^8^ Initial TALEN engineering of CAR19 T cells for T-cell receptor alpha constant (TRAC) and CD52 knockouts (UCART19)^10^ has been extended to other targets including CD20, CD22, and B-cell maturation antigen.^13^^,^^14^ CRISPR/Cas9 editing in the TT52CAR19 products was combined with lentiviral CAR19 expression for multiplexed TRAC and CD52 editing,^11^^,^^15^ and subsequent approaches have combined TCRαβ knockout with site-specific CAR19 insertion into the TRAC locus.^16^, ^17^, ^18^ Subsequently, derivative platforms using CRISPR-guided deamination for multiplexed base-editing have delivered additional “off-the-shelf” CAR T-cell bank iterations which are now being investigated against T-cell acute lymphoblastic leukemia and acute myeloid leukemia.^19^^,^^20^

Although TCRαβ disruption and depletion of residual TCRαβ T cells have effectively addressed the risk of GVHD from mismatched donor T cells, overcoming host-mediated rejection remains a challenge. Augmented lymphodepletion combining chemotherapy and anti-CD52 serotherapy created an advantage for CD52^–^ CAR T cells and delivered potent responses within 28 days, but as anticipated, intense lymphodepletion was associated with viral reactivation, and proceeding to transplant with ongoing viremia increases morbidity.^21^ Next-generation approaches may alleviate such issues through additional engineering steps that remove HLA molecules or promote persistence without the need for intense or protracted lymphodepletion.^22^

Despite high-risk features, sustained remissions beyond 2 years were achieved in 6 of 15 patients, accounting for 40% event-free survival in a nonrandomized, single-arm setting. The results are comparable to published experiences after tisagenlecleucel (autologous CAR19 therapy) in pediatric and young adults with R/R B-ALL.^6^ Most autologous strategies anticipate that CAR T-cell persistence is required for sustained remission, but our experience demonstrates that ∼4 weeks of allo-CAR19 T-cell activity is sufficient for complete disease eradication. Transplantation secured rapid donor-derived reconstitution, including of the B-cell compartment, and all surviving patients received RIC regimens with antithymocyte globulin and low-dose (2-4 Gy) TBI, rather than myeloablative chemotherapy or 12 to 14.4 Gy radiotherapy. Despite small cohorts, our experience suggests that in this setting, intensive transplant preparative regimens were unable to eradicate refractory residual disease.

Besides complete recovery of adaptive immunity and antibody production, another important advantage, a “time-limited” exposure to allo-CAR19 T cells is the amelioration of concerns around genotoxicity risks associated with extended persistence of engineered cells. Transformation events are rare and have to be assessed in the context of patients at increased risks of secondary cancers, with underlying genetic predispositions in addition to vector-related effects.^23^^,^^24^ There is a burden of long-term monitoring for gene-therapy patients, but in the case of allo-CAR19 cells, this has been absorbed into routine post-transplant follow-up.

In summary, although the number of children dosed is relatively small, our longer-term experience in surviving patients indicates that transient exposure to allo-CAR19 T cells achieved deep molecular (PCR-MRD) remissions, and these have been sustained after RIC transplantation with donor-derived recovery of B-cell compartments. The strategy is being refined to reduce the depth and duration of lymphodepletion currently required, including through genome editing of HLA expression and manipulation of pathways promoting T-cell fitness and potency. Ultimately, such “off-the-shelf” CAR T cells could offer cost-effective and accessible alternatives to autologous products for hematological and autoimmune indications.

Patients were treated either under Great Ormond Street Hospital specials license or as part of Research Ethics Committee (REC)-approved studies as follows: TT52CAR19 REC number: 19/LO/1767; and UCART19 PALL REC number: 16/LO/0440.

**Conflict-of-interest disclosure:** Great Ormond Street Hospital has previously received clinical trial support from Cellectis and Servier. W.Q. has consulted for Virocell, Wugen, and AstraZeneca. UCL Business Ltd has filed patents relating to engineered T cells.
