# Supplementary material for: Long-term outcomes of genome-edited “universal” CAR19 T cells for relapsed/refractory B-ALL at a single pediatric center
Source: Blood Adv. 2025 Jul 3;9(18):4750–4. doi: 10.1182/bloodadvances.2025016366 (PMC12496233; doi:10.1182/bloodadvances.2025016366)
Supplement: Supplemental Figures [file BLOODA_ADV-2025-016366-mmc1.pdf]

Supplemental Data

LONG-TERM OUTCOMES FOLLOWING GENOME-EDITED 'UNIVERSAL' CAR19 T-CELLS FOR RELAPSED/REFRACTORY B-ALL AT A SINGLE PAEDIATRIC CENTRE

[1]Daniela Guardo*[1] Avijeet Kumar Mishra*[1] Hebatalla Rashed*[1] Kimberly Gilmour[1] Stuart Adams[1] Danielle Pinner[1] Martin Sauer[2] Ajay Vora[1] Paul Veys[1] Vesna Pavasovic[1] Kanchan Rao[1] Waseem Qasim[1]

[1] Great Ormond Street Hospital for Children NHS Trust & UCL Institute of Child Health, London, UK

[2] Hannover Medical School, Hannover, Germany

Email. W.Qasim@ucl.ac.uk

Supplemental Figure S1

Reactivation of viruses occurred during the period of lymphopenia after lymphodepletion and infusion of allo-CAR19 and was monitored through allo-SCT. Antiviral drug therapy against Cytomegalovirus (CMV) and Adenovirus (ADV) was used as initiated where indicated, and BK virus associated with haemorrhagic cystitis was monitored in urine by serial PCRs.

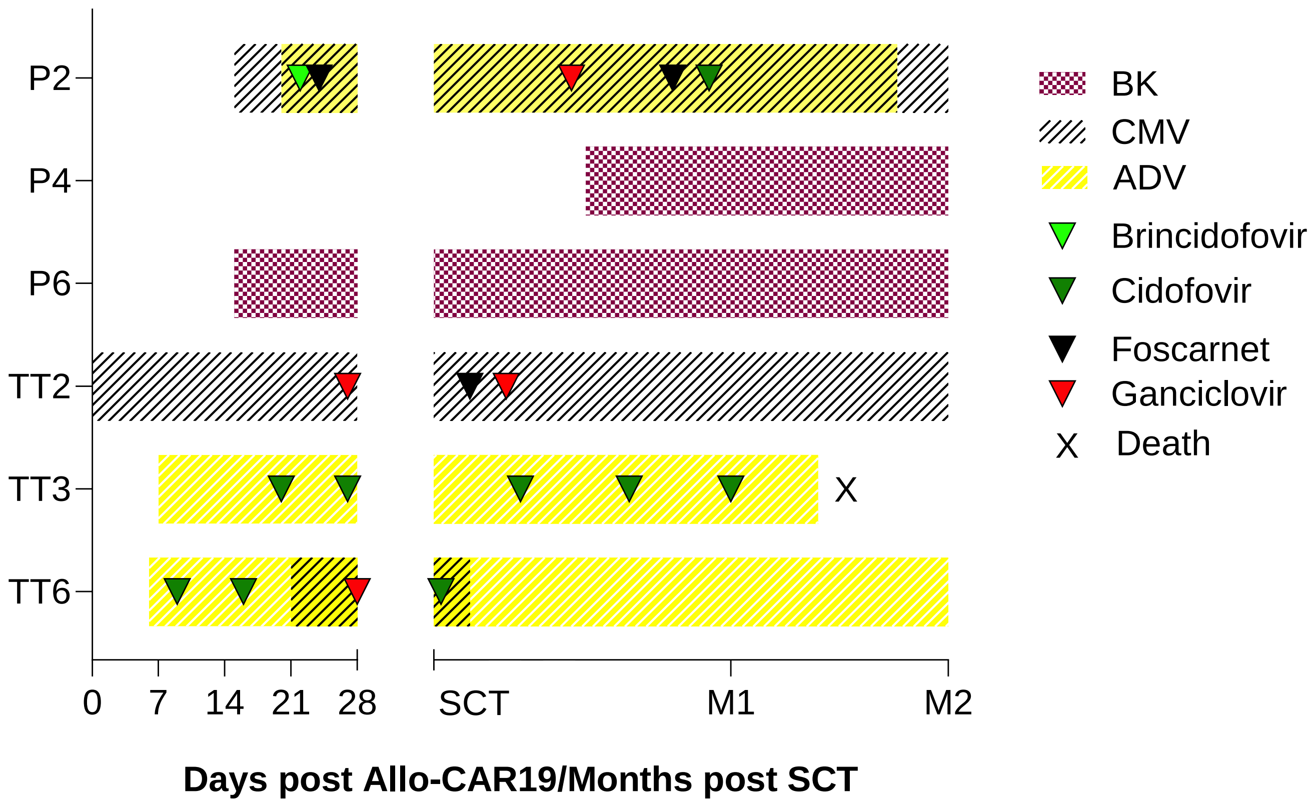

Supplemental Figure S2

Immune reconstitution following allo-SCT in patients who achieved remission after allo-CAR19 infusion. Kinetics of recovery of T cells (CD3) including helper (CD4) T cell subsets, and B cells (CD19) along with IgG,A,M production were as anticipated following standard allo-SCT and removal of residual allo-CAR19 cells during conditioning.

(A) CD3 T cells, (B) CD4 T cells, (C) CD19 B cells, (D) IgG, (E) IgA and (F) IgM levels

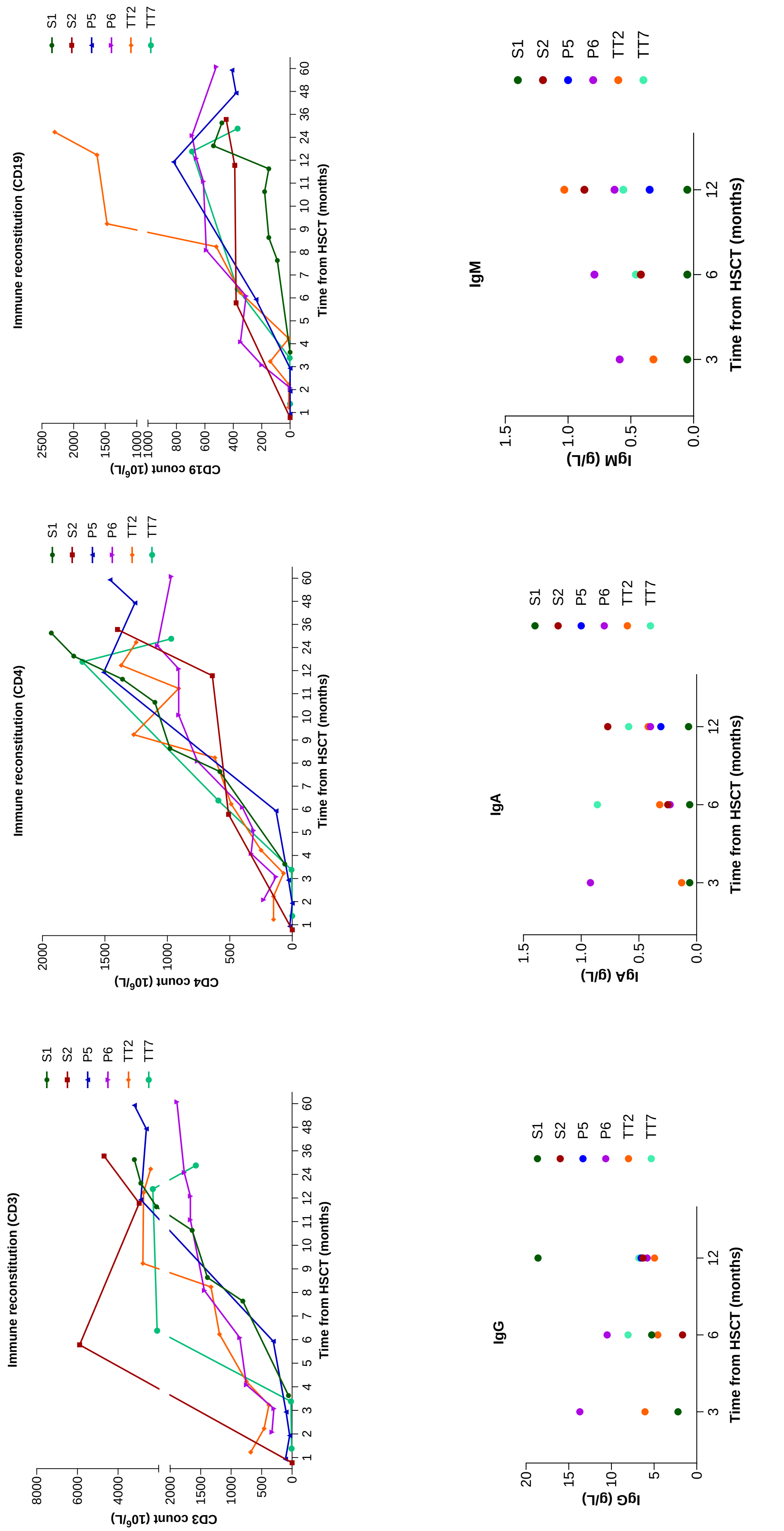
Immune reconstitution (CD3)
CD3 count (10^6/L)
Time from HSCT (months)
Immune reconstitution (CD4)
CD4 count (10^6/L)
Time from HSCT (months)
Immune reconstitution (CD19)
CD19 count (10^6/L)
Time from HSCT (months)
S1
S2
P5
P6
TT2
TT7
IgG
IgG (g/L)
Time from HSCT (months)
IgA
IgA (g/L)
Time from HSCT (months)
IgM
IgM (g/L)
Time from HSCT (months)
